# The effect of 3D virtual surgical planning in sacroiliac joint fusion

**DOI:** 10.1016/j.bas.2025.104334

**Published:** 2025-07-22

**Authors:** Nick Kampkuiper, Asal Abkar, Jorm Nellensteijn, Marjolein Brusse‐Keizer, Gabriëlle Tuijthof, Maaike Koenrades, Femke Schröder

**Affiliations:** a Department of Biomechanical Engineering, University of Twente, Enschede, the Netherlands; b Medical 3D lab, Medisch Spectrum Twente, Enschede, the Netherlands; c Department of Orthopedic Surgery, Medisch Spectrum Twente, Enschede, the Netherlands; d Medical School Twente, Medisch Spectrum Twente, Enschede, the Netherlands; e Health Technology & Services Research, Technical Medical Centre, University of Twente, Enschede, the Netherlands; f Multi-Modality Medical Imaging (M3i) Group, Faculty of Science and Technology, Technical Medical Center, University of Twente, the Netherlands

**Keywords:** Sacroiliac joint fusion, Virtual surgical planning, Simulated fluoroscopy, Implant placement, Patient-specific modeling, Surgical outcomes

## Abstract

**Background:**

Sacroiliac (SI) dysfunction causes of up to 30 % of lower back pain. When conservative treatment is insufficient, SI joint fusion (SIJF) can be indicated to reduce pain. Due to high anatomical variability, poor visibility during intraoperative 2D fluoroscopic imaging, and the absence of 3D spatial information, placing the implant in a stable configuration without damaging critical structures is challenging. To improve patient outcomes, a virtual surgical planning (VSP) method using simulated fluoroscopic images has been developed.

**Research question:**

What is the effect of VSP on patient outcomes, including complications, pain scores, satisfaction scores, and Oswestry Disability Index (ODI) scores?

**Methods:**

This retrospective case-control study compared procedures performed with VSP to those conducted prior to its implementation. Data were collected from the medical records, Numeric Rating Scale (NRS) questions, and patient reported outcome measures (PROMs). All postoperative CT scans were assessed on implant placement (mal)positioning and fractures. Malposition complications were categorized as severe malposition and suboptimal implant placement.

**Results:**

Seventy-eight procedures were included, 43 in the VSP group and 35 in the conventional group. Severe malposition complications reduced from 9 % to 0 % after VSP was implemented. Suboptimal implant placement reduced from 46 % to 9 % of the interventions. Sacral fractures reduced from 37 % to 14 %. All other primary outcome measures did not show a significant difference between the groups.

**Conclusions:**

VSP in SIJF reduced implant malpositioning and sacral fractures. This can lead to better patient outcomes. A larger multicenter study is needed to explore the broader impact of VSP on SIJF.

## Highlights

• Virtual surgical planning (VSP) can improve patient outcomes in sacroiliac joint fusion.
• Severe malposition complications decreased from 9 % to 0 %.
• Suboptimal implant placement decreased from 46 % to 9 %.
• Sacral fractures reduced from 37 % to 14 %.
• Better implant placement can result in superior patient outcomes.

## Graphical abstract

**Figure:**
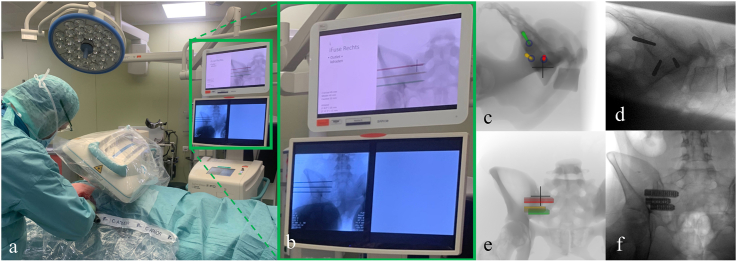

## 1 Introduction

Low back pain is a prevalent condition worldwide with a high socioeconomic impact (Meucci et al., 2015; Hartvigsen et al., 2018). In 10–30 % of the patients the pain is caused by the sacroiliac (SI) joint, also known as SI joint dysfunction (Sembrano and Polly, 2008; Cohen et al., 2013). This can have various causes, such as trauma, previous lumbar fusion surgery, hypermobility syndromes, osteoarthritis, and pregnancy (Cohen et al., 2013; Gartenberg et al., 2021). SI joint dysfunction can be diagnosed by physical examination followed by a confirmatory diagnostic intra-articular injection with local anesthetic. When conservative treatments, such as physical therapy and therapeutic injections, give insufficient response, minimally invasive SI joint fusion (SIJF) can be considered. This can be performed using either a lateral transiliac, posterior intra-articular, or posterolateral transiliac approach with various implants on the market. The lateral transiliac approach using triangular titanium implants is most commonly performed and described (Whang et al., 2023). In this procedure, three implants are inserted through the SI joint to eliminate motion and thereby reduce pain. There is growing evidence supporting the effectiveness of this procedure (Whang et al., 2015, 2023; Sturesson et al., 2016).

SIJF is typically performed using 2D fluoroscopic guidance (Cross et al., 2018). First, the entry points for the guide pins are determined in the lateral view. Once the trajectory of the guide pins is confirmed as safe in inlet and outlet views, the guide pins are inserted to the desired depth. Finally, the implants are placed over the guide pins and the pins are removed.

Although SIJF is generally considered a safe procedure, studies have demonstrated complication rates ranging from 0 % to 17 % (Schoell et al., 2016; Zaidi et al., 2015; Vanaclocha et al., 2018; Shamrock et al., 2019; Heiney et al., 2015). In addition to common surgical complications such as wound infection and hematoma, implant malpositioning can occur, potentially causing nerve root impingement or damage. This complication may lead to neurological complaints such as radiating pain and numbness (Schoell et al., 2016). This complication is prevalent due to high anatomical variability, limited visibility during intraoperative imaging, and the absence of 3D spatial information. Other complications that can occur include fractures of the ilium and/or sacrum. These fractures are more prevalent in sclerotic bone but can also be caused by implant malpositioning (e.g., positioned too close to the cortex). Post surgery long term complications can include non-ingrowing implants or implant loosening (Whang et al., 2019), which may lead to recurring symptoms.

To reduce complications in SIJF, a method for virtual surgical planning (VSP) using simulated fluoroscopic images was developed (Kampkuiper et al., 2023). The VSP is created by virtually positioning the implants in a patient-specific configuration while avoiding critical structures, based on preoperative CT data. Using a 3D model of the pelvis and the position of the implants, simulated fluoroscopic images can be generated to mimic the intraoperative lateral, inlet and outlet views. These images are shown to the surgeon during the intervention, enabling replication of the planned implant positions. This approach is a less costly alternative for navigation-guided techniques. In a previous pilot study, the implant placement accuracy of VSP in ten SIJF procedures was evaluated. The overall mean implant placement accuracy was 4.9 ± 1.26 mm and 4.0 ± 1.44°, and no malposition complications were detected (Kampkuiper et al., 2023). It is expected that accurate replication of the VSP may decrease malposition complications and enhance stability of fusion by placing longer implants in better positions, potentially reducing pain and the likelihood of implant loosening. Using VSP is expected to streamline the procedure as fewer transitions between fluoroscopic views (lateral, inlet, and outlet) are required. This may reduce both radiation exposure and procedure time. However, in the pilot study no comparison to the conventional method was made and complications were only assessed for a small cohort.

Therefore, this study investigates the benefits of VSP in SIJF through a retrospective case-control study in a larger cohort. The primary objective is to investigate the effect of VSP on patient outcomes, including complications, pain scores, satisfaction scores, and Oswestry Disability Index (ODI) (Fairbank and Pynsent, 2000) scores. As a secondary objective, this study assesses the effect of VSP on the procedure time, implant length, radiation time, and radiation exposure.

## 2 Materials and methods

### 2.1 Study design and population

This retrospective single-center case-control study was conducted in accordance with the STROBE (Strengthening the Reporting of Observational Studies in Epidemiology) checklist for observational studies (von Elm et al., 2008). This study compares an intervention group—procedures of patients with SI dysfunction who underwent SIJF with VSP—and a control group—procedures of patients who underwent SIJF without VSP. Primary SIJF procedures between June 2019 and October 2023 were included. Patient selection for SIJF was carefully conducted by the operating orthopedic surgeon. SI dysfunction was confirmed when three or more provocative SI tests (Distraction, Thigh Thrust, Compression, FABER, Gaenslen) and the Fortin Finger Test were positive, followed by a diagnostic intra-articular injection with local anesthetic that resulted in more than 50 % pain reduction (Thawrani et al., 2019).

The VSP method for SIJF was developed and step by step implemented between May and November 2021 (Kampkuiper et al., 2023). After the pilot evaluation of ten cases (Kampkuiper et al., 2023), the method was deemed superior to the conventional method (based on expert opinion), by providing guidance such that the surgeon no longer performed the procedure without VSP (Kampkuiper et al., 2023). Consequently, our hospital adopted VSP for SIJF as standard practice.

All SIJF procedures during the implementation period were excluded. Other exclusion criteria included the use of navigational tools other than the VSP method (for example, intraoperative registration of VSP).

All included procedures consist of primary minimally invasive SIJF with triangular titanium implants using the lateral transiliac approach (iFuse Implant System, SI-BONE, Santa Clara, CA, USA). All procedures were performed by a single surgeon with extensive experience in the operative technique. The follow-up period was at least 6 months. All procedures in the intervention group had a preoperative CT scan. After each procedure a postoperative CT scan was made. In July 2022, the institutional review board approved all clinical evaluation studies where 3D technology was used, including the current study with VSP. Patients who underwent surgery before this date were exempted from informed consent, while those included hereafter gave written informed consent for using their data (nWMO study, K22-24).

### 2.2 Surgical procedures

Patients were positioned in prone position. Using fluoroscopic guidance from a lateral view, the initial entry point for the guide pin was determined.

#### 2.2.1 Control group

**Fig. 1:**
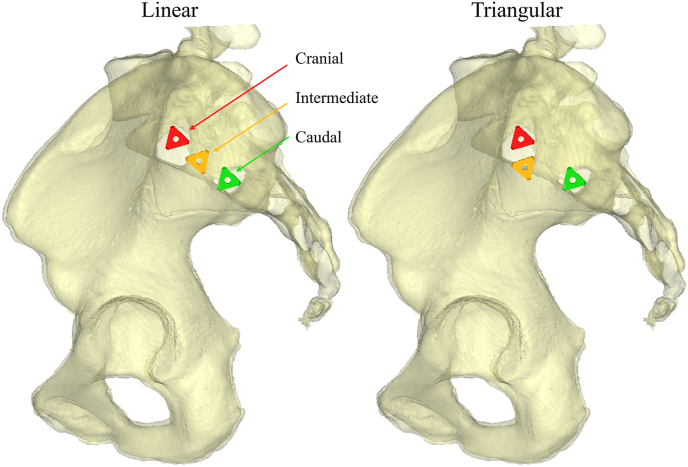
Lateral view on linear and triangular implant configurations. The colours red, orange, and green correspond to the cranial (first), intermediate (second), and caudal (third) implant, respectively. It can be seen that the inter implant distance between the intermediate and caudal implant is increased when a triangular configuration is applied.

All implants were placed parallel to each other and perpendicular to the sagittal plane in a linear configuration (Fig. 1, left view). The cranial implant was generally placed in the S1 segment of the sacrum. The intermediate implant was placed between the S1 and S2 segment. The caudal implant was placed in the S2 segment. While placing the implants, a 15 mm inter implant distance was maintained. The guide pin of the cranial implant was placed and safe positioning is confirmed using lateral, inlet and outlet views, before the implant was inserted over the guide pin. The other two implants were then placed sequentially.

#### 2.2.2 Intervention group

**Fig. 2:**
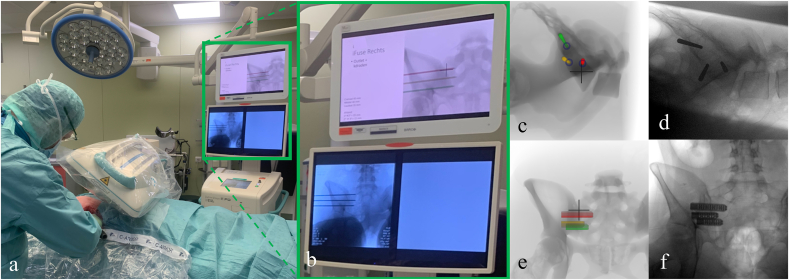
Monitor setup in the operating room. In a, the side-by-side use of the simulated fluoroscopic image and intraoperative image is shown. In b, the two monitors are enlarged. In c and d, examples of the simulated and intraoperative lateral views with guide pins, respectively, are shown. Similarly, in e and f an outlet view with implants is shown. The colours red, orange, and green correspond to the cranial, intermediate, and caudal implant, respectively.

VSP was used in the intervention group. The implant configuration could be adapted to the anatomy of each patient and using a triangular parallel implant configuration (Fig. 1, right view). The cranial implant was generally placed in the cranial part of the S1 segment, the intermediate implant in the caudal part of the S1 segment, and, similar to the conventional method, the caudal implant was placed in the S2 segment. Depending on the anatomy, higher inter-implant distances were applied, most frequently between the intermediate and caudal implant. This triangular approach (right view, Fig. 1), with increased inter-implant distance, allows for better implant positioning with increased stability (Lindsey et al., 2018; Freeman et al., 2021). In contrast to the conventional method, all three guide pins were inserted directly using lateral fluoroscopic guidance before confirming safe positioning in inlet and outlet views. To accurately recreate the VSP, simulated fluoroscopic images with guide pins or implants are displayed on a monitor side-by-side with the monitor of the fluoroscopy device (Fig. 2). The VSP was depicted in a slideshow, the surgeon was able to switch between slides of the lateral, inlet, and outlet view. When all three guide pins were considered to be safely positioned, the implants were inserted sequentially over the guide pins. A more detailed description of how a VSP was created and used can be found in Kampkuiper et al. (2023).

### 2.3 Data collection

**Fig. 3:**
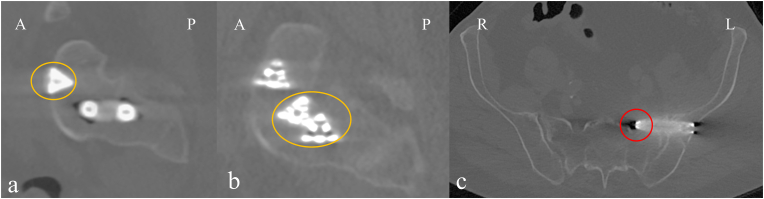
Examples of the assessment of implant malpositioning. In a and b, sagittal slices mainly depict the sacrum, and c is an axial slice containing the entire pelvis. In a, the first implant slightly breached through the anterior cortex of the sacrum (highlighted in orange), but since this patient did not have clinical signs of nerve damage, this is assigned as suboptimal implant placement. In b, an example is shown of a case where the second and third implant made contact. Since this is an unintended outcome but has no clinical consequences, this is also categorized as suboptimal implant placement. In c, a protrusion of the S1 foramen is shown (highlighted in red). This patient experienced radiating pain immediately after surgery and required an immediate repositioning of the implant; therefore, it is categorized as severe malposition complication.

Data were collected from medical records, Numeric Rating Scale (NRS) questions, and patient reported outcome measures (PROMs). Data collected from medical records included baseline patient characteristics, surgical parameters, and complications (Table 1). Intraoperative complications, i.e., implant malpositions and fractures, were assessed on postoperative CT scans by the operating surgeon and a radiologist independently. Discrepancies were discussed, and final consensus was determined by the surgeon and radiologist. Implant malposition was divided into two subcategories: severe implant malposition and suboptimal implant placement. Severe implant malposition was defined as the protrusion of the implant at any location other than the lateral side of the ilium and the SI joint gap leading to nerve impingement and requiring revision surgery, i.e., immediate repositioning of an implant. Suboptimal placement was characterized by cortical breaches that did not cause clinical issues or nerve impingement, as well as instances where two implants made contact. Fig. 3 shows examples of implant malpositioning. Iliac and sacral fractures were distinguished as separate variables due to their distinct etiologies. Iliac fractures often depend upon bone density, whereas sacral fractures can also be attributed to the positioning of the implants. Even small hairline fractures were considered. Postoperative complications, such as hematoma, thrombosis, and wound infection, were collected from the medical records. Radiological implant loosening was confirmed when one or more implants show radiolucency around the implant on postoperative X-ray or CT imaging more than 3 months postoperatively.

**Table 1:** Data collection from medical records.

Baseline characteristics	Surgical parameters	Complications
Age at surgery	Implant lengths	Implant malpositioning
Height	Procedure time	Fractures
Weight	Radiation time	Implant loosening
BMI	Radiation exposure	Infection
Gender		Hematoma
ASA classification		Thrombosis
Smoking status		Neuropraxia
Etiology of SI dysfunction		
Duration of symptoms		
Follow-up time		

Abbreviations: BMI (Body Mass Index), ASA (American Society of Anaesthesiologists), SI (sacroiliac).

To assess pain, satisfaction, and ODI scores, all patients were contacted in April 2024. To avoid recall bias, preoperative (baseline) scores were not collected. Patients rated their current pain at the level of the SI joint on the operated side using the NRS on a scale from 0 to 10, with 0 indicating no pain and 10 the worst possible pain. Furthermore, patients assessed their satisfaction with the surgical outcome using the NRS, where 0 indicated very unhappy and 10 indicated very satisfied. The patients were asked to fill out the ODI questionnaire. Scores ranged from 0 % to 100 %, indicating no disability to most severe disability (i.e., completely bed-bound) (Fairbank and Pynsent, 2000).

### 2.4 Statistical analysis

Statistical analysis was performed using IBM SPSS Statistics 28.0. Baseline characteristics were summarized using descriptive statistics, with categorical variables reported as frequencies with percentages and continuous variables as mean ± SD or median with quartile 1 and quartile 3, depending on distribution.

Differences in primary outcomes (implant malpositioning, fractures, pain scores, satisfaction scores, and ODI scores) and secondary outcomes (procedure time, mean implant length, radiation time, and radiation exposure) between the intervention group and the control group were assessed using Chi-square tests, and, if necessary, a Fisher's Exact test for categorical variables, and T-tests or Mann-Whitney U tests for continuous variables. Risks estimates (odds ratios) were calculated for implant malpositioning and fractures by logistic regression analysis. Values of *p* < 0.05 were considered significant.

To assess a potential learning curve in the consecutively performed procedures, sensitivity analyses were conducted by excluding the first ten cases of the control group and the intervention group.

## 3 Results

**Fig. 4:**
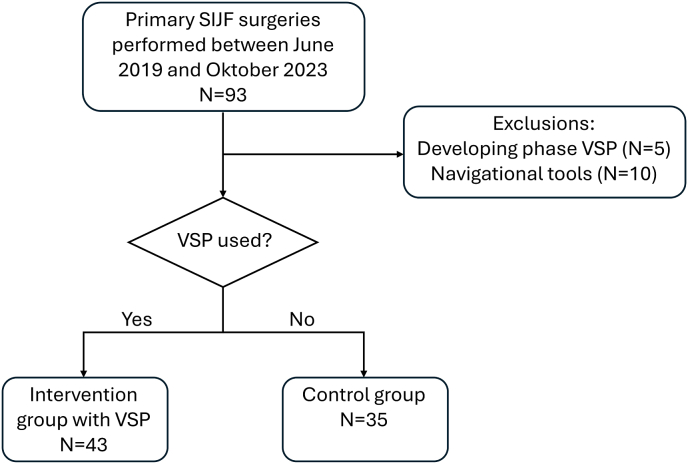
Flow diagram of the inclusion process. Sacroiliac joint fusion (SIJF) procedures that were performed during the development of the virtual surgical planning (VSP) method were excluded, i.e. the developing phase. Other exclusion criteria included the use of navigational tools other than the VSP method.

Sixty-eight patients were included in this study, with in total 78 procedures with 43 in the VSP group (intervention group) and 35 conventional procedures (control group). An overview of the inclusion and exclusion is shown in Fig. 4. The baseline characteristics are shown in Table 2. Only ASA classification and follow-up time differed significantly between the groups. There were relatively fewer ASA 1 and 2 scores but more ASA 3 scores in the intervention group compared to the control group. The control group had a longer follow-up time compared to the intervention group. One patient in the conventional group received two implants; in all other cases, three implants were placed.

**Table 2:** Baseline characteristics.

	Control (n = 35)	Intervention (n = 43)	P-value
Age (years; median [Q1, Q3])	39 [34, 48]	42 [34, 51]	0.442
Height (m; median [Q1, Q3])	1.7 [1.6, 1.8]	1.7 [1.6, 1.8]	0.829
Weight (kg; median [Q1, Q3])	82.0 [73.8, 92.0]	80.0 [68.0, 90.0]	0.297
BMI (kg/m^2^; mean ± SD)	29.0 ± 4.6	28.1 ± 5.7	0.469
Gender (n, %)			1.000
Female	33 (94.3)	40 (93.0)	
Male	2 (5.7)	3 (7.0)	
ASA (n, %)			**0.020**
I	9 (26.5)	5 (11.6)	
II	24 (70.6.)	28 (65.1)	
III	1 (2.9)	10 (23.3)	
Smoking (n, %)			0.383
Yes	9 (25.7)	15 (34.9)	
No	26 (74.3)	28 (65.1)	
Etiology (n, %)			0.126
Trauma	4 (11.4)	6 (14.0)	
Pregnancy	22 (62.9)	15 (34.9)	
Lumbar fusion	2 (5.7)	3 (7.0)	
Hypermobile∗	1 (2.9)	2 (4.7)	
Idiopathic	6 (17.1)	17 (39.5)	
Duration of Symptoms (n, %)			0.073
< 1 year	0 (0)	4 (9.3)	
1–2 years	5 (14.3)	1 (2.3)	
2–5 years	8 (22.9)	8 (18.6)	
> 5 years	22 (62.9)	30 (69.8)	
Follow-up time (years; median [Q1, Q3])	3.6 [3.0, 4.2]	1.5 [1.0, 2]	**<0.001**

Values are given with mean, and standard deviation (SD), median with quartile 1 (Q1) and quartile 3 (Q3), or number with percentage (n, %). ∗ Patients with sacroiliac (SI) dysfunction are often hypermobile, but cases where solely hypermobility, e.g. Ehler-Danlos syndromes, were the cause are categorized in this etiology. Abbreviations – BMI: Body Mass Index, ASA: American Society of Anesthesiologists. Missing data: For one intervention in the control group the ASA classification was missing*.*

Regarding the primary outcomes, significant differences in the frequency of implant malpositioning were observed. Severe implant malposition occurred in 9 % of the cases in the control group compared to 0 % in the intervention group (Table 3). Suboptimal implant placement occurred in 46 % of the control group and 9 % of the intervention group (Table 3), representing an approximately ninefold reduction in risk (OR: 0.11; 95 % CI: 0.04–0.42). Although numerically more severe implant malpositionings were observed in the control group, this was not statistically significant (*p =* 0.086). The incidence of iliac fractures (34 % vs 19 %) was also numerically decreased when using VSP; however, this difference was not significant (Table 3). The incidence of sacral fractures (37 % vs. 14 %) decreased significantly when using VSP (Table 3), corresponding to an approximately fourfold reduction in risk (OR: 0.27; 95 % CI: 0.09–0.83). Three patients experienced temporary neuropraxia in the lower extremity, one patient had a superficial wound infection treated by antibiotics, one patient had an infected hematoma treated by surgical irrigation and antibiotics, and two patients had implant loosening (both diagnosed approximately two years after surgery). All these complications occurred in the conventional group.

**Table 3:** Primary and secondary outcomes of conventional treatment versus treatment using virtual surgical planning (VSP).

		Control N = 35	Intervention N = 43	Odds ratio (95 % CI)	P-value
Primary outcomes	**Implant malpositioning**	19 (54.3)	4 (9.3)	0.09 (0.03–0.030)	**<0.001**
	Severe implant malposition (n,%)	3 (8.6)	0 (0)	NA	0.086
	Suboptimal implant placement (n,%)	16 (45.7)	4 (9.3)	0.11 (0.04–0.42)	**<0.001**
	**Iliac fracture** (n,%)	12 (34.3)	8 (18.6)	0.43 (0.16–1.24)	0.115
	**Sacral fracture** (n, %)	13 (37.1)	6 (14.0)	0.27 (0.09–0.83)	**0.018**
	**Pain score** (NRS; median [Q1, Q3])	4.8 [2.0, 6.5]	3.0 [1.0, 6.0]		0.108
	**Patient satisfaction** (NRS; median [Q1, Q3])	9.0 [7.0, 10]	9.0 [8.0, 10]		0.452
	**ODI score** (0–100; mean ± SD)	33.7 ± 18.5	30.6 ± 16.5		0.494
Secondary outcomes	**Procedure time** (min; mean ± SD)	39.2 ± 7.3	39.7 ± 7.2		0.752
	**Mean implant length** (mm, median [Q1, Q3])	48.3 [46.7, 50.0]	48.3 [46.7, 50.0]		0.645
	**Radiation time** (sec; median [Q1, Q3])	36.0 [29.5, 53.5]	28.5 [25.8, 34.3]		**0.003**
	**Radiation exposure** (cGy·cm^2^; mean ± SD)	1253.4 ± 555.0	1039.9 ± 370.5		0.064

Values are given with mean, and standard deviation (SD), median with quartile 1 (Q1) and quartile 3 (Q3), or number with percentage (n, %). In the case of implant malpositioning and fractures, the frequency of the is noted with a maximum of one per intervention. Abbreviations – CI: Confidence interval, NA: Not applicable, ODI: Oswestry Disability Index, NRS: Numeric Rating Scale. Missing data: pain score 9 % vs. 9 %, satisfaction 9 % vs. 5 %, ODI 23 % vs. 26 %, radiation time 17 % vs. 12 %, and radiation exposure 17 % vs. 12 % for the complete control group and the intervention group, respectively*.*

Pain scores and ODI scores were more numerically favorable for the VSP group compared to the control group, although the differences were not significant (Table 3). There were no statistical differences for the satisfaction scores between both groups (Table 3).

Regarding the secondary outcomes, only radiation time showed a significant decrease from 36 [29.5, 53.5] to 28.5 [25.8, 34.3] seconds when using VSP (Table 3).

Regarding the primary outcomes (implant malpositioning, fractures, pain scores, satisfaction scores, and ODI scores), no substantial differences were observed after excluding the first ten cases from either the control or intervention group (Supplementary Data I). For the secondary outcomes, only slight numerical differences were noted in the sensitivity analysis for the control group for radiation time (control: 36.0 [29.5, 53.5] vs. control excluding first 10 cases: 32.0 [28.8, 55.3]) and radiation exposure (control: 1253.4 ± 555.0 cGy cm^2^ vs. control excluding first 10 cases: 1093.0 ± 452.6 cGy cm^2^).

## 4 Discussion

This study retrospectively investigated the influence of VSP in SIJF on patient outcomes, including complications, pain, satisfaction, and ODI scores by comparing interventions where VSP was used with a control group. Regarding the complications, the prevalence of implant malpositioning—severe malpositions and suboptimal placements—and sacral fractures were significantly lower in the VSP group. There were no significant differences in pain, patient satisfaction, and ODI scores, although numerically, the VSP group scored slightly better for pain and ODI scores. As secondary objectives, the effect of VSP on the procedure time, implant length, and the use of radiation exposure were investigated. Only radiation time showed a significant decrease, indicating that fewer fluoroscopic images were taken after VSP was implemented.

The prevalence of implant malpositioning was significantly reduced in the VSP group, indicating that VSP made SIJF safer in our hospital. In this study, implant malpositioning was defined as severe implant malpositioning and suboptimal placement to thoroughly assess the implant placement. Suboptimal implant placement has not been described in the literature; however, we considered it important to also include this measure because it reflects the quality of implant placement and the potential risk of severe implant malpositioning and/or fractures. When assessing the implant malpositioning in detail, we observed that both the prevalence of severe malpositioning and suboptimal placements dropped. There was a numerical drop from 9 % to 0 % for severe malpositioning and a significant drop from 46 % to 9 % for suboptimal placements. Two reviews found a prevalence of 1.6 % (Shamrock et al., 2019) and 2.1 % (Heiney et al., 2015) for nerve root impingement; this term is comparable to the severe implant malposition rate in this study. This suggests that the prevalence of severe malpositioning in the control group of this study is higher than what is reported in the literature. Yet, there are studies with higher rates of severe implant malposition. For example, the study by Rudolf reported nerve root impingement requiring retraction of an implant in revision surgery in three out of 50 (6 %) cases (Rudolf, 2012). It might be coincidental that in the present study a relatively higher prevalence was observed. Nevertheless, the decrease to zero indicates that VSP has added value in preventing complications. Furthermore, the suboptimal placements could be reduced by 80 %, which indicates that the implants are better positioned with VSP. Fewer suboptimal placements might result in fewer fractures, as placing the implants too close to the cortex might increase the risk of fracturing. Similarly, the chances on severe malposition complications with nerve impingement might also be reduced. Some minor protrusions may still occur in particular cases with narrow anatomy.

The significant decrease in sacral fractures (37 %–14 %) shows that this minor complication is largely attributable to implant placement. The numerical decrease in iliac fractures (34 %–19 %) may indicate that VSP also influences this fracture, although not significantly in this study. Remarkably, fractures in SIJF are barely mentioned in literature; both of the previously mentioned reviews reported two fractures of the ilium in 819 and 432 interventions, respectively (Shamrock et al., 2019; Heiney et al., 2015). Considering the large difference between literature and the fact that in this study a radiologist critically assessed all postoperative CT images, it is most likely that the appearance of small fractures was more critically assessed compared to other studies. Nevertheless, this study shows that the use of VSP resulted in better implant placement, as reflected in the decreased occurrence of fractures.

Other types of complications, such as neuropraxia, hematoma, wound infections, and implant loosening occurred less frequently in the VSP group but the prevalences are too small to draw conclusions. Theoretically, VSP could influence neuropraxia, as better guide pin placement could allow for fewer guide pins to be placed close to neural structures. Differences in the prevalence of hematomas and wound infections was not expected, mainly because adding VSP does alternate the size of the incision and still the same instruments are used for implantation. Furthermore, in theory, better implant positioning could result in increased stability and thus lower risk of implant loosening.

The numerical differences for pain and ODI scores suggest that VSP may provide added value. These differences might be the result of superior implant positioning, i.e., creating a better fusion of the SI joint due to better implant distribution and increased implant depth into the sacrum. A meta-analysis by Whang et al. found similar ODI scores as in the control group of this study (Whang et al., 2023). However, the NRS pain score was slightly lower in their study (Whang et al., 2023). The NRS score for patient satisfaction of 9.0 [7.0, 10] and 9.0 [8.0, 10], for the control and VSP groups, respectively, demonstrate that most patients were satisfied with the outcome.

Regarding the secondary outcomes, the significant decrease in radiation time indicates that using VSP results in 21 % fewer intraoperative fluoroscopic images. Remarkably, radiation exposure did not differ significantly. The reduction in radiation exposure might be less prominent because there could be a slight increase in lateral fluoroscopic images. Lateral images require more radiation because X-rays must penetrate more tissue in this view compared to inlet and outlet views. Hence, as a result of automated dose control the device uses more radiation in the lateral view. The surgeon may perform more lateral images because placement in the lateral view is crucial for replicating the preoperative plan.

Procedure time and implant length did not differ significantly in this study. Nevertheless, this study shows that VSP, unlike other more complex navigation-guided systems (Shuman et al., 2022), does not increase the overall procedure time. The implant length did not differ between both groups. However, the use of VSP allowed the surgeon to change the implant configuration from a linear to a triangular approach. This way, the implants can be better positioned across the SI joint, offering better stabilization (Lindsey et al., 2018; Freeman et al., 2021). However, there is no method yet to quantify this better positioning of the implants.

There could be two ways to further reduce the radiation exposure and procedure time while using 2D fluoroscopic guidance and VSP. Namely, superimposing the VSP onto intraoperative imaging using a 3D to 2D registration method, would make it possible to place the implants with more ease and eliminates the necessity of exactly replicating the simulated fluoroscopic images (Lankheet et al., 2024). Another option could be to develop a minimally invasive patient specific guide that allows placing all three guide pins simultaneously.

The sensitivity analyses suggests that a potential learning curve had no impact on the primary outcomes of this study. Only slight differences in radiation time and exposure were observed in the control group when the first ten cases were excluded. Therefore, while the results for radiation time and exposure should be interpreted with some caution, the absence of differences in the primary outcomes indicates that the observed effects are likely attributable to VSP itself, rather than to procedural experience gained over time.

Another potential advantage of VSP is the increase in surgeon confidence. Although this was not assessed in the current study, while this is difficult to define and measure in SIJF, it is expected that VSP in SIJF has similar benefits to those observed in other preoperative applications of virtual 3D models (Hyde et al., 2019; Locketz et al., 2017).

The strengths of this study include the assessment of multiple outcome parameters, including complications and other patient outcomes (pain, ODI, and satisfaction scores). Additionally, surgical parameters such as implant length, procedure time, radiation time, and radiation exposure were assessed to provide a comprehensive understanding of the effect of VSP in SIJF. Another strength of this study is the objective assessment of the complications in postoperative CT scans, as all scans were assessed by an independent radiologist.

A limitation of the study is the differences in the baseline characteristics for ASA classification and follow-up time. Due to the low number of events we were not able to correct for this using a multivariate logistic regression analysis. However, these differences do not affect the outcomes of implant malpositioning and fractures, as these are primarily influenced by surgical precision. If there were an influence, it is expected to be an underestimation of VSP because the intervention group had a higher percentage of ASA 3, indicating that the general health of some patients was worse. Furthermore, the shorter follow-up might result in that some patients in the intervention group (follow-up <1 year) might not have reached the full benefit of the procedure. The difference in follow-up could influence implant loosening (n = 2 in the control group vs. n = 0 in the intervention group). Therefore, this cannot be compared between groups. For ODI and radiation related outcomes, there was approximately 25 % and 15 % missing data in both groups, respectively (Table 3). Since this was almost equal in both groups, it is expected that this did not influence the outcomes. Another limitation of this study lies in its retrospective design. The response rate to pain and ODI scores at standardized intervals was low, resulting in a lack of preoperative baseline and follow-up data for both groups. Consequently, we had to contact all patients to rate their current pain and ODI scores, which led to a difference in follow-up time between the groups. We considered performing a prospective (randomized) study to facilitate optimal comparison between the groups. However, we deemed this ethically inappropriate, as the surgeon expressed reluctance to perform procedures without VSP following its implementation. A note to mention is that before the implementation date of VSP, the solid iFuse implants were replaced with 3D-printed versions in March 2021. Approximately two-thirds of the control group have solid implants implanted. It is expected that the change in implants did not influence the results, because a post-market surveillance study found similar adverse event rates (Cher et al., 2018) and a prospective trial found similar patient outcomes (including pain, ODI and satisfaction scores) (Patel et al., 2020). The only difference lies in the proportion of bone bridging at 12 months (3D printed 70 % vs. solid 45 %) (Patel et al., 2020). Hence, the 3D printed implants allow for faster fusion and might lower the chance of implant loosening in the short term (<2 year). Since our control group, with mostly solid implants, has a follow up of at least 2.5 years, there is no difference expected between the study groups. Still, this is why implant loosening could not be analyzed in this cohort. Furthermore, it is a limitation that it is a single-center study with one surgeon performing the procedure. Lastly, due to relatively low prevalence of certain complications, e.g. severe implant malposition and ilium fractures, there was insufficient power to detect a significant difference between the groups. Similarly, this study might also lacked power to confirm differences for pain and ODI scores.

Therefore, it is recommended to perform a larger multi-center prospective study. Implementation of VSP in SIJF is relatively easy without the need for additional (costly) hardware. The only requirement could be a training in how to use VSP, including how to mimic the simulated image with the intraoperative fluoroscopic image. Nevertheless, the results of this study suggest that VSP has significant added value in SIJF since it made the procedure safer. Therefore, it is assumed that VSP might also benefit other surgeons, especially in cases with difficult anatomy, such as narrow SI joints or dysmorphic sacra.

## 5 Conclusion

In this single surgeon retrospective study, implementing VSP in SIJF significantly improved the procedure's safety by reducing implant malpositioning and sacral fractures. This decreased implant malpositioning and sacral fractures indicate better implant placement, which can result in superior patient outcomes. To confirm these results a multi-center study is required.

## Declaration of competing interest

The authors declare that they have no known competing financial interests or personal relationships that could have appeared to influence the work reported in this paper.
