# Supplementary material for: The effect of 3D virtual surgical planning in sacroiliac joint fusion
Source: Brain Spine. 2025 Jul 22;5:104334. doi: 10.1016/j.bas.2025.104334 (PMC12318284; doi:10.1016/j.bas.2025.104334)
Supplement: Multimedia component 1 [file mmc1.docx]

| *Table S1. Sensitivity analysis control group vs. control group excluding the first ten cases.* | | | |
| --- | --- | --- | --- |
|  |  | **Control (All Cases)**  **N=35** | **Control (Excl. First 10)**  **N = 25** |
| Primary outcomes | **Implant malpositioning** | 19 (54.3) | 13 (52.0) |
|  | Severe implant malposition (n,%) | 3 (8.6) | 2 (8.0) |
|  | Suboptimal implant placement (n,%) | 16 (45.7) | 11 (44.0) |
|  | **Iliac fracture** (n,%) | 12 (34.3) | 9 (36.0) |
|  | **Sacral fracture** (n, %) | 13 (37.1) | 10 (40.0) |
|  | **Pain score** (NRS; median [Q1, Q3]) | 4.8 [2.0, 6.5] | 5.0 [2.0, 6.6] |
|  | **Patient satisfaction** (NRS; median [Q1, Q3]) | 9.0 [7.0, 10] | 9.0 [6.8, 10] |
|  | **ODI score** (0-100; mean ± SD) | 33.7 ± 18.5 | 31.3 ± 19.1 |
| Secondary outcomes | **Procedure time** (min; mean ± SD) | 39.2 ± 7.3 | 38.2 ± 6.48 |
|  | **Mean implant length** (mm, median [Q1, Q3]) | 48.3 [46.7, 50.0] | 48.3 [46.7, 50.0] |
|  | **Radiation time** (sec; median [Q1, Q3]) | 36.0 [29.5, 53.5] | 32.0 [28.8, 55.3] |
|  | **Radiation exposure** (cGy·cm²; mean ± SD) | 1253.4 ± 555.0 | 1093.0 ± 452.6 |
| *Values are given with mean, and standard deviation (SD), median with quartile 1 (Q1) and quartile 3 (Q3), or number with percentage (n, %). In the case of implant malpositioning and fractures, the frequency of the is noted with a maximum of one per intervention. Abbreviations - ODI: Oswestry Disability Index, NRS: Numeric Rating Scale.* | | | |

| *Table S2. Sensitivity analysis intervention group vs. intervention group excluding the first ten cases.* | | | |
| --- | --- | --- | --- |
|  |  | **Intervention (All Cases)**  **N=43** | **Intervention (Excl. First 10)**  **N = 33** |
| Primary outcomes | **Implant malpositioning** | 4 (9.3) | 2 (6.1) |
|  | Severe implant malposition (n,%) | 0 (0) | 0 (0) |
|  | Suboptimal implant placement (n,%) | 4 (9.3) | 2 (6.1) |
|  | **Iliac fracture** (n,%) | 8 (18.6) | 6 (18.2) |
|  | **Sacral fracture** (n, %) | 6 (14.0) | 4 (12.1) |
|  | **Pain score** (NRS; median [Q1, Q3]) | 3.0 [1.0, 6.0] | 3.0 [1.0, 5.5] |
|  | **Patient satisfaction** (NRS; median [Q1, Q3]) | 9.0 [8.0, 10] | 9.0 [8.0, 10] |
|  | **ODI score** (0-100; mean ± SD) | 30.6 ± 16.5 | 32.0 ± 15.9 |
| Secondary outcomes | **Procedure time** (min; mean ± SD) | 39.7 ± 7.2 | 38.0 ± 7.6 |
|  | **Mean implant length** (mm, median [Q1, Q3]) | 48.3 [46.7, 50.0] | 48.3 [46.7, 50.0] |
|  | **Radiation time** (sec; median [Q1, Q3]) | 28.5 [25.8, 34.3] | 29.0 [25.5, 33.5] |
|  | **Radiation exposure** (cGy·cm²; mean ± SD) | 1039.9 ± 370.5 | 967.0 ± 367.6 |
| *Values are given with mean, and standard deviation (SD), median with quartile 1 (Q1) and quartile 3 (Q3), or number with percentage (n, %). In the case of implant malpositioning and fractures, the frequency of the is noted with a maximum of one per intervention. Abbreviations - ODI: Oswestry Disability Index, NRS: Numeric Rating Scale.* | | | |
